# Exploring the Digital Mental Health Literacy of the Tunisian population: A Cross-sectional Online Survey

**DOI:** 10.1192/j.eurpsy.2024.1137

**Published:** 2024-08-27

**Authors:** O. Chehaider, M. Lagha, A. Adouni, I. Ben Romdhane, W. Homri, R. Labbane

**Affiliations:** ^1^Psychiatry C; ^2^Razi hospital, Manouba, Tunisia

## Abstract

**Introduction:**

In the digital age, the landscape of mental health information dissemination and consumption in Tunisia has experienced a profound transformation. As the digital revolution continues to reshape our lives, understanding how individuals seek and interact with mental health information online has become increasingly critical.

**Objectives:**

The primary objectives of this study are as follows:

- To comprehensively investigate the digital mental health literacy of individuals in Tunisia by administering an insightful online questionnaire.
- To delve into the multifaceted aspects of how Tunisians engage with mental health content on digital platforms, unveiling their comfort levels, preferences, and decision-making factors.

**Methods:**

This study conducted an online survey comprising three sections. The first gathered demographic information to profile our diverse participants. The second explored internet usage patterns, unveiling their digital activities. The third delved into perceptions of mental health information on social media, revealing preferences. Our survey reached participants of various ages and locations in Tunisia.

**Results:**

The findings of this study cast a revealing spotlight on the digital mental health landscape in Tunisia. A significant proportion of our respondents frequently engaged with various social media platforms. Notably, Instagram emerged as the favored platform for 80% of our participants, while 72% chose Facebook as their preferred digital sanctuary. Intriguingly, 57% of our respondents actively embarked on quests for mental health information on YouTube, with a distinct preference for video-based content.

In the labyrinth of online mental health information, our participants exhibited a discerning eye. They assigned paramount importance to source credentials, references to reputable sources, and unwavering adherence to established medical guidelines. However, beneath this discernment, a noteworthy 65% harbored doubts regarding the accuracy of online information, reflecting the inherent challenges and complexities of navigating the digital information ecosystem.

Furthermore, our study unearthed areas where social media platforms may still grapple with shortcomings in addressing the multifaceted needs of mental health consumers. Participants eloquently expressed concerns about the accuracy of information, the availability of reliable platforms, and the crucial need for a diverse array of perspectives in mental health content on social media.

**Conclusions:**

This study offers key insights into Tunisia’s digital mental health landscape. It highlights prevalent digital information consumption and preferences. Emphasizing the need for credible and diverse mental health information on social media is vital. This sample lays the foundation for enhancing available content, better supporting mental well-being in Tunisia.

**Disclosure of Interest:**

None Declared

